# Breast Cancer Mortality in Young Women in Brazil

**DOI:** 10.3389/fonc.2020.569933

**Published:** 2021-01-25

**Authors:** Juliana Dalcin Donini E. Silva, Rosana Rosseto de Oliveira, Mariana Teixeira da Silva, Maria Dalva de Barros Carvalho, Raissa Bocchi Pedroso, Sandra Marisa Pelloso

**Affiliations:** ^1^ Health Sciences Department, Graduate Program in Health Science, State University of Maringá, Maringá, Brazil; ^2^ Health Sciences Department, Graduate Program in Nursing, State University of Maringá, Maringá, Brazil

**Keywords:** young women, breast neoplasm, trend analysis, epidemiology, mortality

## Abstract

**Objective:**

Malignant breast cancer is the leading cause of death by cancer in young women. The study aimed to determine if breast cancer mortality among young women has increased between the period from 1996 to 2017 in Brazil.

**Methods:**

A time-series analysis of breast cancer mortality rate in young women (20–39 years old) was carried out. Mortality data, from 1996 to 2017, were collected from the Mortality Information System of the Health Ministry, and demographic data, from the Brazilian Institute of Geography and Statistics. Trends in mortality were performed by Joinpoint Regression, the spatial distribution of the mortality rate was done with the QGIZ Software version 2.18, and Spearman’s correlation coefficient was used to correlate the mortality rates with the Human Development Index.

**Results:**

There was an increase in breast cancer mortality rates in young women in the majority of Brazilian states, with an upward trend in all regions. The correlation with the Municipal Human Development Index, income, and education had a significant impact on the mortality rate for women from 30–39 years old in both time frames evaluated and for women from 20–29 years old, only from 1996 to 2000.

**Conclusion:**

The data obtained in the study, showed that even though the breast cancer mortality rate of young women is lower than women over 40 years old, it has been increasing in all regions of Brazil, mostly for women from 30–39 years old, suggesting that this group should be included in screening programs.

## Introduction

Malignant breast cancer is the most common type of cancer in women, except for non-melanoma skin cancer. In Brazil, it is estimated in 2020, 66,280 new cases of women with breast cancer, representing 29.7% of all types of cancer with an incidence of 43.7 per 100 thousand women. As for mortality, in 2017, there were 16,724 deaths of women with breast cancer, corresponding to a death risk of 16.1 per 100 thousand (1).

In young women (20–39 years old), breast cancer is also the most prevalent type of cancer, as well as the leading cause of death by cancer in most countries, and it is considered a problem that is still little discussed and studied, because when compared to the incidence and mortality in the age group over 40 years, the numbers are significantly lower (2).

The incidence of breast cancer in young women has shown a significant increase. In the Brazilian capitals of Porto Alegre (1993 and 2005) and Goiânia (1998 to 2008), there was a considerable increase of it in the 20 to 39 age group (3). Likewise, in the United States, it was observed a growth from 24.6 to 31.7 per 100 thousand women in the same age group, in 1975 and 2015, respectively (4).

In young women, breast cancer occurs heterogeneously, with worse prognosis and high mortality, and in more advanced stages, presenting with larger and more aggressive tumors (5). A study carried out in the United Kingdom showed poor survival in women with breast cancer under the age of 40, even with the modernization of the treatments performed, in addition to the risk of tumor recurrence (6). Compared to older women, the prognosis is worse and the chance of death is greater when diagnosed in stages I and II (7).

In the United States, the estimated breast cancer deaths in women under 40 years old, were 3% of the total deaths from this condition (8). Given this scenario, it is crucial to better understand breast cancer mortality in this age group to submit more effective measures for screening, early identification, and case management.

It was observed that the mortality rate of young women (under 40 years old) has been escalating in Brazil; hence, this study aimed to determine if breast cancer mortality among young women has increased between the period from 1996 to 2017 in Brazil and correlate it with socioeconomic variables.

## Methodology

A time-series evaluation of breast cancer mortality rate in young women in Brazil was conducted with data from 1996 to 2017. The number of deaths related to breast neoplasm (C50) was used according to the 10th Revision of the International Statistical Classification of Diseases and Related Health Problems (ICD-10). The mortality data from the Mortality Information System of the Ministry of Health and demographic data of the IBGE (Brazilian Institute of Geography and Statistics) were obtained at the Department of Informatics of the Ministry of Health (https://datasus.saude.gov.br/).

For the analysis, the data were obtained by geographic regions: North, Northwest, Midwest, Southeast, South, and 26 states. For the mortality rate, a specific mortality rate was calculated for each year and location, using the formula: Number of deaths from breast cancer in the geographic region, divided by the reference population, multiplied by 100,000.

The SDR from breast neoplasms were analyzed as dependent variables (Y-axis) and the years 1996 to 2017 as independent variables (X-axis). Analysis were carried out using the Joinpoint Regression Program, Version 4.7.0.0. (February 2019). There was an increase in the mortality rate when the tendency was of growth and the minimum value of the 95% CI > 0. However, the reduction occurred when there was a decline in the tendency and the maximum value of the 95% CI < 0. Stability was defined when, regardless of the trend, 95% CI included the value of 0. The average annual percentage changes for particular mortality rates were described with the 95% confidence interval. Statistical hypotheses were verified at the significance level α = 0.05.

A correlation analysis was used to verify the existence of a relationship between breast cancer mortality rates. For this analysis, the 5-year periods from 1996 to 2000 and 2013 to 2017 and the human development index (HDI) of 2000 and 2010, respectively, were chosen (9). The year 1996 was chosen because it was the year of the beginning of the registration of cases of breast cancer deaths in the Mortality Information System and 2017 because it was the last year of registration updated at the time of data collection.

The HDI follows the three dimensions assessed worldwide: income, longevity and education. In this work we use the HDI, HDI income and HDI education. It is through the HDI that countries are classified as developed, developing or underdeveloped.

Spearman’s correlation coefficient was used for the analysis, which were interpreted according to the following parameters: if correlation coefficient is <0.4, the correlation is considered to be of low magnitude, if correlation coefficient is ≥0.4 to <0.5 the correlation is considered to be moderate magnitude and, finally, correlation coefficient of ≥0.5 represents a strong correlation. The significance level of 5% was considered. For the analysis, the software Statistical Package for the Social Sciences (SPSS), version 20.0 was used.

The cartographic base of Brazil that contains the borders of the States is publicly available online in shapefile (SHP) on the website of the Brazilian Institute of Geography and Statistics (IBGE). Color maps were created to demonstrate the distribution of overall breast cancer mortality rates in the age groups 20–29 and 30–39 years in the Brazilian states. All figures were designed using QGIS version 2.8. The spatial distribution of breast cancer rates was presented in the years 1996 and 2017, displayed in pink scales, in which the darkest shades illustrate the highest rates and the lightest shades the lowest rates.

The data collected are available in the public domain, with no need for authorization from the Committee of Ethics and Research with Human Beings.

## Results

**Figure 1:**
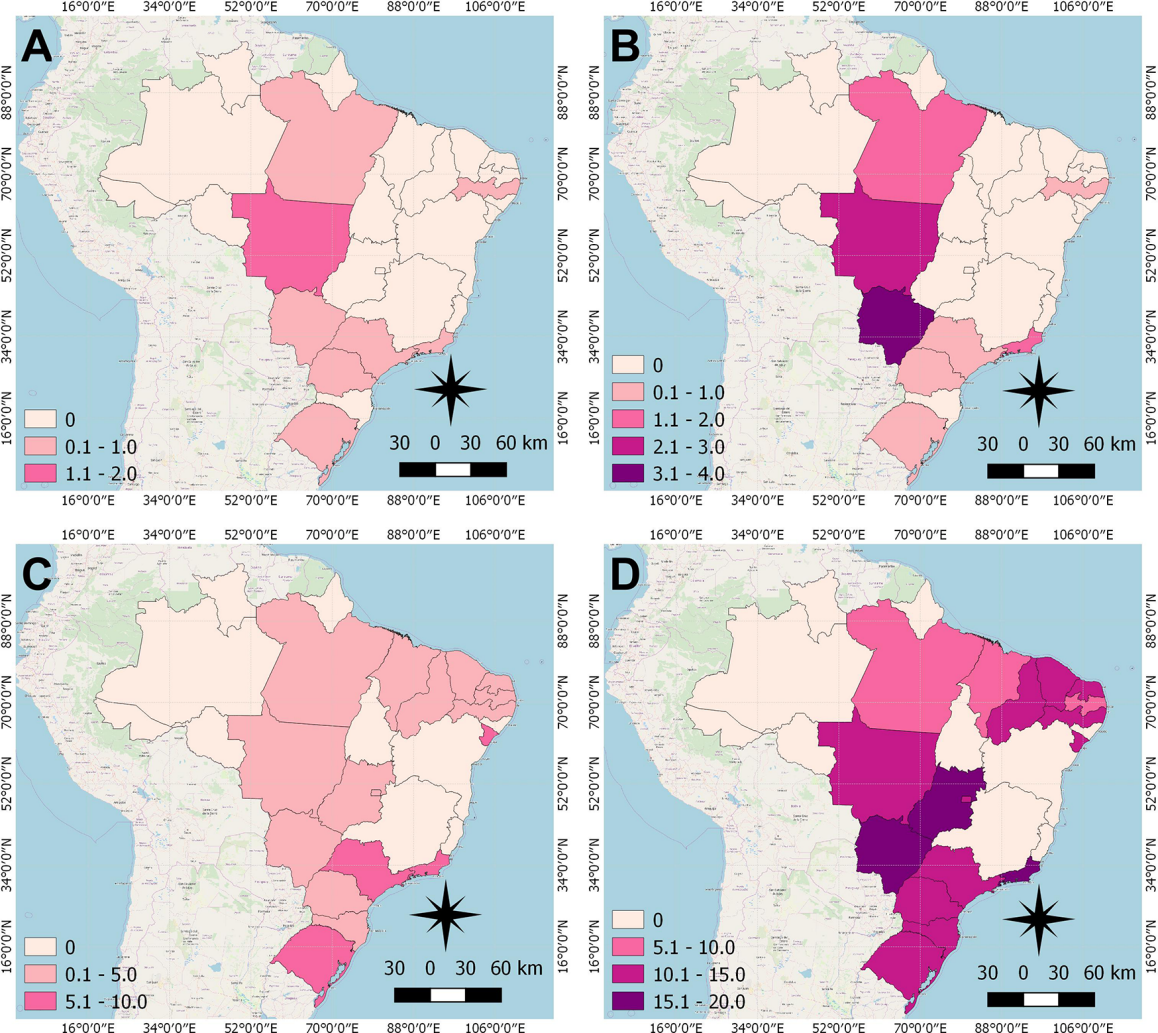
Distribution of Breast cancer mortality rate of women under 40 years of age in Brazilian states, in the years 1996 and 2017. **(A)** 20–29 years, 1996; **(B)** 20–29 years, 2017; **(C)** 30–39 years, 1996; **(D)** 30–39 years; 2017.

From 1996 to 2017, there were 19,105 deaths of young women with breast cancer in Brazil. According to the spatial distribution of the mortality rates, in all Brazilian states, there was a raise for women under 40 years old (**Figure 1**). This increase was observed in the states of Pará, Mato Grosso, Mato Grosso do Sul and Rio de Janeiro, for women from 20–29 years old in 1996 (**Figure 1A**) to 2017 (**Figure 1B**). For the age group of 30–39 years, there was an increase in most states, and rates remained constant only in 10 states (**Figures 1C, D**).

**Figure 2:**
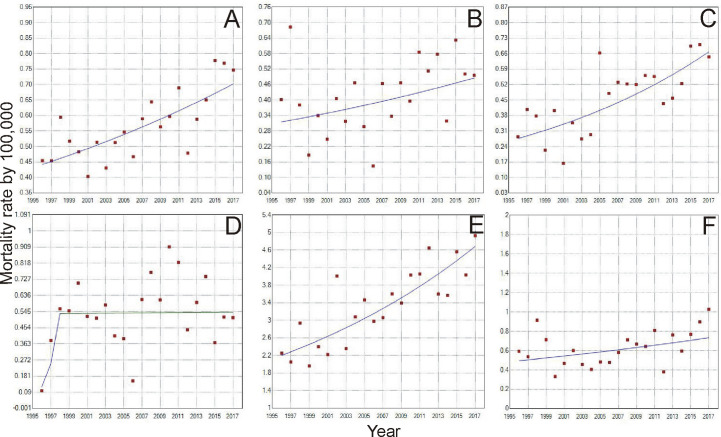
Trends of Breast Cancer Mortality rate from 1996 to 2017, of women aged 20-29 years in Brazil **(A)**. North region **(B)**, Northeast region **(C)**, Midwest region **(D)**, Southeast region **(E)** and South Region **(F)**. [D] Period: 1996-1998; APC: 110.7; CI: -36.2 – 595.2; p=0.2; Period: 1998-2017; APC: 0.1; CI: -3.4 – 3.7; p=1.0.

According to the Joinpoint Regression (**Tables 1** and **2**), the mortality rate of young women with breast cancer in Brazil has been escalating. For women from 20–29 years old, there was an increase of 2.2% a year from 1996 to 2017 (**Figure 2A**). When we analyze the five regions of Brazil, for this age range, the state of Pará, in the North region, had an increase in the mortality ratev of 5.7% a year (**Figure 2B**). The whole Northeast region had an increase of 4.3% (**Figure 2C**), in the Midwest region, Mato Grosso and Mato Grosso do Sul state, increased in 3.4 and 3.1% a year, respectively (**Figure 2D**). The whole Southeast region had an increase of 1.9% a year (**Figure 2E**), with São Paulo and Rio de Janeiro state, being 1.7 and 2.6%, respectively. There were no significant values for the South region (**Figure 2F**), even though there was an increase in the mortality rate.

**Table 1:** Trends of Breast cancer mortality in women under 39 years of age in Brazilian states from 1996 to 2017.

	Age 20-29					Age 30-39				
	MR 1996	MR 2017	APC	IC	P	MR 1996	MR 2017	APC	IC	P
**Brazil**	0.45	0.75	2.2	1.3 – 3.1	<0.05	4.79	12.42	4.6*	2.7 – 6.5	<0.05
**North**	0.40	0.50	2.1	-0.5 – 4.7	0.1	2.25	4.93	3.7	2.6 – 4.8	<0.05
Acre	–		–	–		–	–	–	–	
Amapá	–		–	–		–	–	–	–	
Amazonas	–		–	–		–	–	–	–	
Pará	0.60	1.79	5.7	4.1 – 7.3	<0.05	2.06	9.30	6.5	4.4 – 8.6	<0.05
Rondônia	–	–	–	–		–	–	–	–	
Roraima	–	–	–	–		–	–	–	–	
Tocantins	–	–	–	–		–	–	–	–	
**Northeast**	0.28	0.64	4.3	2.3 – 6.3	<0.05	3.18	5.29	2.9	2.0 – 3.6	<0.05
Alagoas	–	–	–	–		–	–	–	–	
Bahia	–	–	–	–		–	–	–	–	
Ceará	–	–	–	–		3.03	10.11	5.7	4.0 – 7.5	<0.05
Maranhão	–	–	–	–		0.64	7.02	10.5	8.0 – 13.2	<0.05
Paraíba	–	–	–	–		0.46	8.33	16.9*	10.6 – 23.5	<0.05
Pernambuco	0.45	0.26	-2.5	-5.6 – 0.6	0.1	4.40	13.38	4.0	1.9 – 6.2	<0.05
Piauí	–	–	–	–		0.58	11.51	11.6	8.1 – 15.3	<0.05
Rio Grande do Norte	–	–	–	–		4.94	12.91	8.4	5.6 – 11.3	<0.05
Sergipe	–	–	–	–		8.06	13.69	6.9	3.5 – 10.5	<0.05
**Midwest**	0.10	0.51	7.4*	-3.7 – 19.9	0.2	3.88	7.18	1.3	0.1 – 2.4	<0.05
Goiás	–	–	–	–		4.50	16.29	7.1*	2.7 – 11.6	<0.05
Mato Grosso	1.12	2.30	3.4	0.3 – 6.5	<0.05	3.65	11.46	3.4*	-2.0 – 9.0	0.2
Mato Grosso do Sul	0.56	3.99	3.1	0.2 – 6.0	<0.05	2.00	16.86	4.4	0.9 – 8.0	<0.05
Distrito Federal	–	–	–	–		4.53	10.14	2.5	-1.2 – 6.2	0.2
**Southeast**	0.59	0.83	1.9	0.8 – 3.0	<0.05	5.88	7.25	0.7*	0.1 – 1.3	<0.05
São Paulo	0.45	0.86	1.7	0.1 – 3.2	<0.05	6.21	13.17	4.4*	3.0 – 5.8	<0.05
Rio de Janeiro	0.77	1.18	2.6	1.2 – 4.1	<0.05	7.20	17.87	5.5*	2.9 – 8.2	<0.05
Espírito Santo	–	–	–	–		–	–	–	–	
Minas Gerais	–	–	–	–		–	–	–	–	
**South**	0.59	1.03	1.9	-0.1 – 3.9	0.1	5.478	6.33	0.7*	-0.4 – 1.8	0.2
Paraná	0.61	0.66	1.1	-1.7 – 3.9	0.4	4.74	11.78	4.3*	-2.3 – 11.2	0.2
Santa Catarina	–					3.98	13.29	4.5	2.7 – 6.5	<0.05
Rio Grande do Sul	0.77	1.42	1.7	-1.4 – 4.8	0.3	6.88	12.29	3.1*	0.1 – 6.1	<0.05

MR, Mortality rate per 100.000; APC, Annual percent changes calculated by Joinpoint Regression Analysis; [*] AAPC, Average Annual Percent Change calculated by Joinpoint Regression Analysis; CI, confidence interval.

**Table 2:** Descriptive values for the Annual Percentage Change of the regions and states that presented Average Annual Percent Change calculated by Joinpoint regression.

	Age 20-29				Age 30-39			
	MR	APC	CI	P	MR	APC	CI	P
**Brazil**								
Brazil (1996–2011)	–	–	–	–	4.79–5.62	0.8	0.3–1.4	<0.05
Brazil (2011–2014)	–	–	–	–	5.62–10.66-	28	13.0–44.9	<0.05
Brazil (2014–2017)	–	–	–	–	10.66–12.42	2.7	-3.5–9.2	0.4
**Northeast**								
Paraíba (1996–1998)	–	–	–	–	0.46–2.26	119.2	20.2–299.9	<0.05
Paraíba (1998–2017)	–	–	–	–	2.26–8.33	9.4	7.5–11.4	<0.05
**Midwest**								
Midwest (1996–1998)	0.10–0.56	110.7	-36.2–595.2	0.2	–	–	–	
Midwest (1998–2017)	0.56–0.51	0,1	-3.4–3.7	1.0	–	–	–	
Goiás (1996–2011)	–	–	–		4.50–4.64	0.7	-2.6–4.1	0.7
Goiás (2011–2017)	–	–	–		4.64–16.29	9.4	9.4–42.3	<0.05
Mato Grosso (1996–2003)	–	–	–		3.65–0.98	-11.8	-23.3–1.5	0.1
Mato Grosso (2003–2017)	–	–	–		0.98–11.36	11.9	6.5–17.5	<0.05
**Southeast**								
Southeast (1996–2005)	–	–	–		5.88–5.30	-0.9	-2.1–0.3	0.1
Southeast (2005–2017)	–	–	–		5.30–7.25	1.9	1.2–2.7	<0.05
São Paulo (1996–2010)	–	–	–		6.21–5.68	-0.6	-1.8–0.6	0.3
São Paulo (2010–2017)	–	–	–		5.68–13.17	15.2	11.3–18.3	<0.05
Rio de Janeiro (1996–2007)	–	–	–		7.20–7.71	1.0	-1.4–3.3	0.4
Rio de Janeiro (2007–2017)	–	–	–		7.71–17.87	15.2	7.8–23.1	<0.05
**South**								
South (1996–2005)	–	–	–		5.47–4.56	-1.3	-3.3–0.7	0.2
South (2005–2017)	–	–	–		4.56–6.33	2.2	0.9–3.5	<0.05
Paraná (1996–2011)	–	–	–		4.74–5.39	1.3	-0.6–3.2	0.2
Paraná (2011–2014)	–	–	–		5.39–14.93	39.0	-10.0–114.8	0.1
Paraná (2014–2017)	–	–	–		14.93–11.78	-9.6	-27.3–12.3	0.3
Rio Grande do Sul (1996–2007)	–	–	–		6.88–4.62	-4.2	-8.0–0.1	<0.05
Rio Grande do Sul (2007–2017)	–	–	–		4.62–12.89	11.7	6.5–17.1	<0.05

MR, Mortality rate per 100.000; APC, Annual percent changes calculated by Joinpoint Regression Analysis calculated by Joinpoint Regression Analysis; CI, confidence interval.

**Figure 3:**
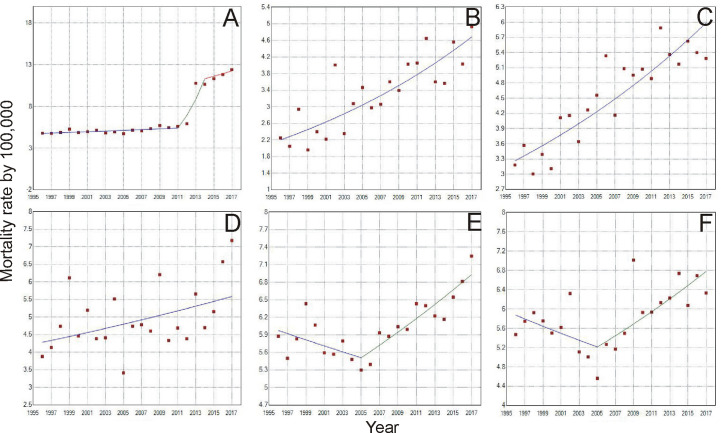
Trends of Breast Cancer Mortality rate from 1996 to 2017, of women aged 30-39 years in Brazil **(A)**, North region **(B)**, Northeast region **(C)**, Midwest region **(D)**, Southeast region **(E)**, and South Region **(F)**. **(A)** Period: 1996 - 2011; APC: 0.8; CI: 0.3–1.4; p =<0.05; Period: 2011-2014; APC: 28.0; CI: 13.0–44.9; p =<0.050; 2011-2017; APC: 2.7; CI: -3.5-9.2; p =0.4; =0.3; **(E)** Period: 1996-2005; APC: -0.9; CI: -2.1–0.3; p =0.1; Period: 2005-2017; APC: 1.9; CI: 1.2–2.7; p =<0.05 **(F)** Period: 1996-2005; APC: -1.3; CI: -3.3–0.7; p =0.2; Period: 2005–2017; APC: 2.2; CI: 0.9–3.5; p = <0.05.

When analyzing the mortality rate trend for the age group of 30 to 39 years (**Tables 1** and **2**), there was an increase of 4.6% a year in Brazil (**Figure 3A**). For the North region, there was an increase of 3.7% a year (**Figure 3B**), and Pará was the only state in this region with a record of death in this age group, with 6.5 deaths/100 thousand women. In the Northeast, the increase was 2.9% (**Figure 3C**), with the state of Paraíba standing out with 16.9%, followed by Piauí (11.6%) and Maranhão (10.5%). In the Midwest, in general, the growth was 1.3% (**Figure 3D**), being higher in the state of Goiás with 7.1%. In the Southeast and South regions, the lowest growth rates were observed (**Figures 3E, F**, respectively), with 0.7% in each region, whereas in the Southeast, the state of Rio de Janeiro had the highest growth of 5.5% and in the South, the state of Santa Catarina obtained 4.5% between 1996 and 2017.

Still concerning the time trend of young women mortality due to breast cancer, some points of statistically significant increase or decrease were identified, as can be observed for the age group of 30 to 39 years where, in Brazil, from 2011 to 2014 there was a 28% variation in the mortality rate (**Figure 3A**). In the Southeast, between 1996 and 2005, there was a downward trend of -0.9%, followed by an increase of 1.9% between the years 2005 and 2017 (**Figure 3E**). Similarly, from 1996 to 2005, there was a downward trend of mortality in the South region of -1.3% and followed by an increase of 2.2% in the years 2005 to 2017 (**Figure 3F**).

When correlating breast cancer mortality in young women with sociodemographic indexes (**Table 3**), the municipal human development index (MHDI), the Income HDI and Education HDI had a significant impact in the mortality rate for women from 30–39 years old in both periods evaluated and for women from 20–29 years old, only in 1996 to 2000.

**Table 3:** Correlation between breast cancer mortality rates and Municipal Human Development Index, according to age group.

1996-2000	MHDI		HDI Income		HDI Education	
	Correlation coefficient*	*p*	Correlation coefficient*	*p*	Correlation coefficient*	*p*
20–29 years	0.54	0.004	0.60	0.001	0.50	0.008
30–39 years	0.68	<0.001	0.66	<0.001	0.67	<0.001
**2013**–**2017**						
20–29 years	0.25	0.210	0.21	0.297	0.20	0.313
30–39 years	0.53	0.004	0.509	0.007	0.508	0.007

MHDI, Municipal Human Development Index; HDI, Human Development Index; *Spearman’s correlation coefficient.

## Discussion

In oncology, there is no common ground about the age group that specifies “young women”, however, in the literature; most publications refer to young women, those under 40 years of age. Thus, for this study, the age group of 20 to 39 years was selected to be evaluated (10).

Breast cancer in young women is still poorly studied and there are a lot of uncertainties about the specific characteristics of the pathology, such as prognosis, recurrences, and mortality (11). The prognosis for young women is worse than for women aged 50 to 69 years, being related to late presentation and more aggressive tumor biology. The late diagnosis stands out the importance of community and health professionals that pays attention to complaints related to the breasts. Regarding that the tumor biology is aggressive, there are still several questions, lacking the intensification of research in the same degree of importance of women with triple-negative cancer (12).

With the escalating incidence and consequently, an increase in the mortality rate that rises with age is a concern because of the few existing studies.

In this study, we chose to include only cases of death from breast cancer coded as ICD10–C50, registered in the Ministry of Health’s Information System, since the correction of mortality rates from ill-defined causes can overestimate mortality rates, especially in places where there are more deaths from ill-defined causes.

Breast cancer mortality in young women has increased in the last two decades in Brazil, its regions and states, presented rates higher in 2017 when compared to 1996 for the two age groups studied. The regions with the highest APC was the Midwest, with 7.4% for women between 20–29 years old and North, with APC of 3.7% for women between 30–39 years old.

Rocha-Brischiliari et al. (13) also described increasing mortality rates in young women (20–49), in all regions of Brazil but they described that the Northeast region was the one with the largest increase and Southeast and South regions with the highest average rates from 1996 to 2013. This gap could be due to the 5 years difference among data (2013 to 2017) and that women from 41 to 49 years old were included.

Balmant et al (14). described that breast cancer mortality rates, in Brazil and its regions, in the 2009–2013 period, for adolescents and young adults, were higher for the 25–29 years old range in all regions (above 4.4 per million). Nevertheless, these data reinforces that there has been an increase in mortality of young women, from breast cancer in Brazil, and that it has been happening differently in all regions.

A survey carried out on countries in Oceania and the Americas among women aged 20 to 49, from 2002 to 2012, pointed out that there was an increase in the mortality rate, due to breast cancer, in Brazil, Colombia, Venezuela, and the Philippines, while for the other countries, the trend was to have decreased rates (15).

In the United States, the American Cancer Society reveals that breast cancer is the second leading cause of death by cancer. The chance of an American woman dying from breast cancer is 2.6%, and since 2007, rates have remained stable in women under 50 years of age. However, between the years 2013 and 2017, there was a reduction in the annual rate of 1.3% per year and a significant improvement in the survival of young women with breast cancer, attributing this reduction to early diagnosis, improved awareness, and adequate treatment (4, 16).

Similarly in Chile, from 1995 to 2013, there was a downward trend in breast cancer mortality rates of 0.8% a year for women from 30 to 39 years old. This decrease is the result of implemented strategies such as prevention, early diagnosis, timely treatment, efficient measures that seek to reduce breast cancer mortality, and improve life quality (17).

While the mortality rates in low and middle-income countries are increasing, in high-income countries, the opposite has been observed. In several high-income countries, breast cancer mortality rates have decreased, mainly due to advances in treatment. However, there are still divergences in middle and low-income countries (15).

In France, even though the incidence of breast cancer for young women increased by 1.1% per year from 1990 to 2018, mortality decreased by 1.3% in the same period, corresponding to 5% of deaths inyoung women (10). Similar to Shanghai in China where the trend was for decreasing mortality rates due to this condition (18). Also, a trend analysis of breast cancer mortality in women aged 30 to 39 years from 1996 to 2009 was carried out in Switzerland, showing a downward trend from three to 1.6 deaths per 100 thousand women (19).

In this sense, there is interference from socioeconomic level in the actions and adherence of women to breast cancer prevention measures. As for education, the higher, the better the search for help, when women understand and make themselves understood in health services (20).

In this study, it was possible to verify a positive correlation between the municipal human development index (MHDI), the HDI Income and Education in the HDI, which has a significant impact on the mortality rate of women aged 30–39 years.

The HDI follows three pillars: income, longevity and education. In this work we use the general HDI, the income HDI and the education HDI. It is through the HDI that countries are classified as developed, developing or underdeveloped. The measure is from zero to one, the closer to one, the better the HDI.

Although Brazil is economically considered a high-middle income country, the HDI is considered high and reflects breast cancer mortality rates, as shown in **Table 3**. The positive correlation of mortality in Brazil with the HDI means that the higher the HDI, the greater the chance of dying from breast cancer.

For the 20–29 age group in the 2013–2017 5-year period, there was no correlation with the MHDI, we believe that a more detailed study could reveal this phenomenon with greater precision. However, it must necessarily be related to an improvement in data recording, raising mortality rates and distancing the MHDI variable as a factor related to the increase in deaths.

Delays of diagnosis and initiation of treatment are the main aspects that determine the poor prognosis in this age group. In Brazil, the average time between diagnosis and the start of treatment was 59 days, with variation between regions, the longest recorded in the South and Southeast, with 61 and 65 days respectively, remembering that the recommendation is not to exceed 60 days (21). Another study carried out in Singapore showed that the delay of more than 90 days for the beginning of the treatment can interfere in the survival of women with invasive cancer (22).

In addition to the delay in starting treprognosisatment after diagnosis, we can add the delay in diagnosis after the identification of the first symptoms. A study carried out in Brazil identified that the delay between the signs and symptoms and the diagnosis was on average 102 days. Therefore, the delay of diagnosis and the delay in starting treatment can negatively reflect on survival and mortality. The delay in diagnosis can occur in two ways, the first related to the user’s delay in seeking care after identifying the signs and symptoms of breast cancer, and the delay related to the health system, including problems with scheduling appointments and diagnostic tests, interfering with the initiation of therapies (23).

One of the most common ways of identifying breast cancer is screening, which is carried out through the mothers’ clinicians and imaging tests, such as mammography and ultrasound. The most effective and that really shows an effect in reducing mortality is screening by mammography, however, there is no recommendation to perform it in women under 50 years old (24).

Although there are no studies that prove the effectiveness of clinical breast examination in reducing mortality from breast cancer in young women, it is still a strategy that can be easily performed by a medical professional or nurse, in addition to having a low cost. Clinical breast examination is usually performed opportunistically, a factor that justifies the low coverage of this form of screening, according to a study carried out in Brazil in which half of the women do not undergo a clinical breast examination (23).

If carried out in an organized manner, with awareness campaigns and through programs, it can reach a larger number of women, benefiting the age group of young women who are not part of the mammographic screening. A study carried out in Indonesia showed that clinical breast examination is a viable alternative for screening, since underdeveloped countries have difficulty maintaining a mammographic screening program (25).

Still, there is evidence that preventive measures should be used, including health education with incentives to change lifestyle, reducing alcohol and tobacco consumption, maintaining adequate weight and physical activity. The diagnosis in the shortest possible time and initiation of appropriate treatment improves prognosis, increasing chances of cure and reduced mortality from breast cancer in young women (26).

One of the limitations of the study was to use secondary data, in which gaps were identified in the database, where there was no record of cases of death in some states, especially in the age group of 20 to 29 years. In view of this limitation, it appears that there are possible flaws in the information about the deaths that have occurred, incurring a limitation in the planning of preventive actions, diagnosis and treatment. The fact that mortality from breast cancer in young women is lower in relation to other age groups, does not diminish the concern and the impact on public health.

Considering the gap verified in the reported data, mainly in the age group of 20 to 29 years old, the need for the commitment of Brazilian municipalities and states in the systematization of health information is highlighted, since the lack of data brings losses to the planning of actions and health management in some Brazilian states.

## Conclusion

In 2017, the World Health Assembly presented a cancer prevention and control resolution through an integrated approach, urging governments and WHO to accelerate actions to achieve objectives specified in the Global Action Plan in order to reduce premature cancer mortality (27). The data obtained in the study, supported these actions, showing that even though the mortality rate of young women is lower than for women over 40 years old, it has been increasing in all regions of Brazil, mainly for women from 30–39 years old, suggesting that controlled actions of prevention and screening by clinical breast examination should be carried out by public managers.

## Data Availability Statement

The raw data supporting the conclusions of this article will be made available by the authors, without undue reservation.

## Ethics Statement

Ethical review and approval was not required for the study on human participants in accordance with the local legislation and institutional requirements. Written informed consent for participation was not required for this study in accordance with the national legislation and the institutional requirements.

## Author Contributions

JS, RP, SP created the project. JS, RP also contributed to the data analysis and writing of the manuscript. JS, RP, RO acquired the original data and developed the methodology. RP, JS, RO, MS, contributed to conceptualize the study and edited the manuscript. RP, SP, MC also supervised the general work. All authors contributed to the article and approved the submitted version.

## Funding

This work was carried out with the support of the Coordenação de Aperfeiçoamento de Pessoal de Nível Superior - Brazil (CAPES) - Financing Code 001; and the Graduate Program in Health Sciences, State University of Maringá (UEM).

## Conflict of Interest

The authors declare that the research was conducted in the absence of any commercial or financial relationships that could be construed as a potential conflict of interest.
